# An Uninformed Decision-Making Process for Cesarean Section: A Qualitative Exploratory Study among the Slum Residents of Dhaka City, Bangladesh

**DOI:** 10.3390/ijerph19031465

**Published:** 2022-01-27

**Authors:** Jesmin Sultana, Ipsita Sutradhar, Musarrat Jabeen Rahman, Abdullah Nurus Salam Khan, Mohiuddin Ahsanul Kabir Chowdhury, Enam Hasib, Charu Chhetri, S. M. Hasan Mahmud, Tahsin Kashem, Sanjeev Kumar, Zaw Toe Myint, Mahbubur Rahman, Tarique Md. Nurul Huda, Shams El Arifeen, Sk Masum Billah

**Affiliations:** 1 Infectious Diseases Division, International Centre for Diarrhoeal Disease Research, Bangladesh (icddr,b), Mohakhali, Dhaka 1212, Bangladesh; mahbubr@icddrb.org (M.R.); tarique.huda@icddrb.org (T.M.N.H.); 2 BRAC James P Grant School of Public Health, BRAC University, Dhaka 1213, Bangladesh; IpsitaEva69@gmail.com (I.S.); mohiuddin.chowdhury@bracu.ac.bd (M.A.K.C.); 3 International Health, Johns Hopkins Bloomberg School of Public Health, 615 N Wolfe St Suite E8527, Baltimore, MD 21205, USA; mrahma28@jhmi.edu; 4 Maternal and Child Health Division, International Centre for Diarrhoeal Disease Research, Bangladesh (icddr,b), Mohakhali, Dhaka 1212, Bangladesh; nurus.salam.aupi@gmail.com (A.N.S.K.); shams@icddrb.org (S.E.A.); billah@icddrb.org (S.M.B.); 5 Family Health International, Dhaka 1212, Bangladesh; EHasib@fhi360.org; 6 Falck Global Assistance, Gurugram 122015, India; charuchhetri51@gmail.com; 7 Bangladesh National Nutrition Council, Mohakhali, Dhaka 1212, Bangladesh; hasan1109nssb@gmail.com; 8 Bangladesh Palliative and Supportive Care Foundation, Dhaka 1212, Bangladesh; tahsin.kashem@gmail.com; 9 Health Systems Transformation Platform, New Delhi 110070, India; snjvkumar386@gmail.com; 10 Health Systems Strengthening, Community Partners International, Yangon 11201, Myanmar; drzawtoemyint@gmail.com; 11 Sydney School of Public Health, The University of Sydney, Sydney, NSW 2006, Australia

**Keywords:** cesarean section, decision-making process, shared decision-making, information asymmetry, Bangladesh

## Abstract

The decision-making process and the information flow from physicians to patients regarding deliveries through cesarean section (C-section) has not been adequately explored in Bangladeshi context. Here, we aimed to explore the extent of information received by mothers and their family members and their involvement in the decision-making process. We conducted a qualitative exploratory study in four urban slums of Dhaka city among purposively selected mothers (*n* = 7), who had a cesarean birth within one-year preceding data collection, and their family members (*n* = 12). In most cases, physicians were the primary decision-makers for C-sections. At the household level, pregnant women were excluded from some crucial steps of the decision-making process and information asymmetry was prevalent. All interviewed pregnant women attended at least one antenatal care visit; however, they neither received detailed information regarding C-sections nor attended any counseling session regarding decisions around delivery type. In some cases, pregnant women and their family members did not ask health care providers for detailed information about C-sections. Most seemed to perceive C-sections as risk-free procedures. Future research could explore the best ways to provide C-section-related information to pregnant women during the antenatal period and develop interventions to promote shared decision-making for C-sections in urban Bangladeshi slums.

## 1. Introduction

Cesarean sections (C-sections) can be life-saving surgery [1] in cases of complicated normal vaginal delivery (NVD) [2]. C-sections also have some advantages for the mother and the baby, such as no labor pain and reduced chances of perineal injury, urinary incontinence, and birth trauma [3]. However, unnecessary C-sections do not add any additional benefit for the mother and the child [2]. In cases of unnecessary C-sections compared to NVDs, hospital stays are longer and there may be increased risks for wound infection, post-discharge urinary tract infection, moderate blood loss, fever, and death among cesarean mothers and low Apgar score and neonatal complications among newborns [4,5,6,7]. The rate of C-sections is very high in high-income countries, and the rate in low- and middle-income countries (LMICs) has been rapidly increasing over time, especially in South Asia [8]. Globally, the C-section rate is 21% [9], and 13% in South and South-East Asia [8]. In 2014, one of every four livebirths in healthcare facilities was through C-section in Bangladesh [10]. In 2019, the rate increased to 36%, and the rate was much higher in urban areas (47.3%) than in rural areas (32.8%) [11]. Almost half of the institutional deliveries in the slums of Dhaka city were by C-section [12]. Clinical reasons for C-sections include maternal hypertension during pregnancy, non-vertex presentation during childbirth, prolonged pregnancy, prolonged labor, amniotic fluid disorder, antepartum hemorrhage, obstructed labor, fetal distress, previous C-section, and severe health problems, including infections during pregnancy [13]. Other non-clinical factors associated with increased rates of C-sections include socioeconomic status, place of residence, education, culture and beliefs, women’s choice, fear of NVD, facility type, provider type, financial profit, workload, and surgical preferences of the provider [8,14,15,16,17,18]. Doctor-patient communication, information flow, and women’s involvement in the decision-making process play a vital role in the final health care choice decision, including C-sections [19,20,21,22,23].

Shared decision-making is a collaborative approach to healthcare-related decision-making between physicians and patients, including their family members [24], and it is a key part of patient-centered care in clinical practice [25]. In shared decision-making processes, physicians share detailed information about the disease process, outcome, available treatment options, and potential risks and benefits of each alternative with the patients and their family members [26]. Then, the patients and their family members make an informed decision based on their thorough understanding of those options [26]. Shared decision-making is a way to reduce information asymmetry in healthcare services [26], whereby a lack of knowledge flow from physicians to patients during service provision can create mistrust in doctor-patient relationships [27,28]. Information asymmetry is often associated with malpractice, dissatisfaction [28], and costly and low-quality services [29]. In high-income countries, despite measures in place to ensure shared decision-making in clinical practice, making shared decisions into routine practice among clinical practitioners remains a challenge [26,27,30,31]. In the context of LMICs, shared decision-making in healthcare services is nearly nonexistent. The provider-dominated healthcare decision in clinical care is one of the barriers to implementing shared decision-making in LMICs [32].

In Bangladesh, where the C-section rate is rapidly increasing, there is little evidence of whether or not the decision to have a C-section is a shared one. Therefore, we conducted a study to explore the decision-making process, health and economic consequences, and coping strategies pertaining to the C-section experiences of Dhaka city slum residents. Mothers who delivered via C-section and their family members shared their experiences during the decision-making process. This paper aims to explore the extent of information related to C-sections received by the Dhaka city slum residents who had experienced cesarean birth and their involvement during the decision-making process.

## 2. Materials and Methods

### 2.1. Study Design and Study Settings

We used a phenomenological research design to address the study objectives [33]. We had three units of analysis in our study: mothers, their husbands, and other family members. We followed hermeneutic approach in this study [33]. This qualitative exploratory study was conducted in four urban slums of Dhaka city in Mirpur (Kollaynpur Porabosti, Vasantek slum, and Mirpur-11 Football Gram Camp) and Gulshan thana (Korail slum) between October 2016 and January 2017 [34].

### 2.2. Sampling Technique

From the list of slums located in Dhaka city, we randomly selected four slums. From each slum, we purposively chose participants who had given birth through C-section or were a family member who was involved in the C-section decision-making process.

### 2.3. Study Participants

The study participants comprised mothers who resided in the selected slums and delivered a baby via C-section within one-year preceding data collection along with their family members. We excluded severely ill individuals and individuals with physical and/or mental impairment.

### 2.4. Data Collection

We chose in-depth interviews as the primary data collection method because it is the best tool to collect experience-related information [35]. We used pretested semi-structured interview guides to conduct the interviews. The mothers and the family members were interviewed using separate interview guides. We interviewed respondents in their preferred place to ensure adequate privacy. Two research teams, each comprised of two graduate-level students (at least one female), from BRAC James P Grant School of Public Health, conducted interviews. We stopped data collection upon reaching data saturation, when no new information emerged from the data.

### 2.5. Data Analysis

After collection, audio-recorded data were transcribed into Bengali verbatim and later translated into English. We then read the transcripts, notes, and memos several times for data familiarization. We adapted the data analysis method of phenomenological studies described by Moustakas [36]. We coded the transcripts manually based on a codebook. The codebook was prepared at the initial stage of the study, and it contained a-priori codes and sub-codes based on pre-determined themes derived from the study objectives. During coding, we also added some emerging themes in the codebook as inductive codes. We then compiled data from each interview in an excel sheet and sorted this by theme. We interpreted the data by summarizing the composite description of each theme based on the compiled data and impressions from notes and memos. We also prepared a frequency matrix that showed the distribution of each code and sub-code that emerged from each interview. Finally, we presented the findings using the summaries of each theme which were used to describe the context or recreate the experiences of the participants. We also presented the data frequency of each theme and relevant quotes which were extracted during coding of interviews.

The codebook contained operational definitions of each of the themes and sub-themes. Some of the codes were: primary decision-maker, household-level decision-maker, and actors’ involvement in the decision-making process. The primary decision-maker was the first person who advised a pregnant woman or her family members to choose C-section, usually the physician. The household-level decision-maker was the family member who gave consent for a C-section based on the primary decision-maker’s suggestion, usually the husband. Actors of the decision-making process, such as physicians, husbands, pregnant women, and family members, had varied levels of consenting power and participation rates throughout the process. Here, we explored consenting power through inquiring if the actors consented to the process, how their consent was valued by other actors, and if their consent influenced the final decision to go through the C-section process. In each case, we graded the actors’ involvement “less” when s/he had no or less consenting power than the other actors in the decision-making process.

### 2.6. Ethical Considerations

We received ethical approval from the Ethical Review Committee of BRAC James P Grant School of Public Health, BRAC University, Mohakhali, Dhaka 1213, Bangladesh, to conduct the study. We obtained informed written consent from participants to conduct interviews and audio-records. We maintained confidentiality and anonymity of respondents and responses during data collection, analysis, and sharing of the results.

## 3. Results

### 3.1. Sociodemographic Characteristics of the Sample

We interviewed 19 respondents, of which seven were mothers who had given childbirth through C-section, five were the husbands of these mothers, five were mothers-in-law of these mothers, and two were the mothers’ other family members. The age range of participants who delivered via C-section was 19–40 years. Husbands’ ages ranged from 28–35 years, and the ages of mothers-in-law and other family members ranged from 40–60 years old. Most of the female respondents (12 out of 14) were homemakers. The husbands’ reported occupations were electrician, small business owner, auto-rickshaw driver, and salaried job. The mothers had completed 3–8 years of formal education, and husbands had completed 7–12 years. However, mothers-in-law and other family members had no formal education. The household’s monthly income was 6000–45,000 BDT (~76–573 USD, in 2016).

### 3.2. Involvement of Mothers and Family Members in Decision-Making

According to five mothers, three husbands, four mothers-in-law, and two other family members, 14 out of 19 respondents, most of the time the mothers had a minimal role in the C-section decision-making. They were informed about the necessity of a C-section by the physicians, but mothers were not asked about their opinions or involved in any decision-making-related discussions. During the interviews, all respondents referred to C-sections as Caesars.

One 19-year-old mother stated that:

*During the time of childbirth, we were in [an urban healthcare center] for two days … Everyone was coming and going, but the childbirth was delayed in my case. Everyone was saying that my childbirth would be late … the doctors checked my condition many times … then my mother got angry at the hospital and phoned my husband, and my husband said, enough! We are waiting for a long time, no need to wait anymore … they did the ultra (ultrasonography), blood tests, and then did the Caesar … My husband did not talk to me when all these things were going on.*

Most of the respondents (five mothers, three husbands, five mothers-in-law, and two other family members (15 out of 19 respondents)) mentioned that the physicians were the primary decision maker about the mode of childbirth. Pregnant women were advised for C-sections during antenatal care (ANC) visits or when the pregnant women were in labor.

One 34-year-old husband explained:

*Suddenly my mother-in-law noticed that my wife was not looking good, so she said to visit the doctor for checkups. After the visit, the doctor said, no, the condition is bad; there will be complications if we do not do the operation today. I again went to another hospital, visited a private doctor… She also said, there will be a problem if my wife is not operated today.*

In most cases (three mothers, five husbands, two mothers-in-law, 10 out of 19 respondents), the husbands were actively involved during decision-making. They decided to go for C-sections at the household level, communicated with physicians and family members, arranged financial support, coordinated with other family members, and managed caregivers to ensure post-surgery support for the mothers.

A 35-year-old husband said:

*I took my wife to the doctor. I did the checkup every month to see if there was any problem with my children … It was I who told about the Caesar. As there were two babies, that’s why I wanted to do the Caesar without taking the risk. I arranged everything for the Caesar … We went to [a tertiary level government hospital] according to the date … She (the pregnant woman) became sicker after going there. The doctors told us that the Caesar needed to be done right then. They didn’t have a doctor to perform Caesar that night. They said, doctors don’t stay here at night, here doctors stay all day but not at night. Later I hired an ambulance from there to take her to [another tertiary level government hospital] … we had our babies doing the Caesar at [another tertiary level government hospital] at 4 am.*

Respondents also reported the involvement of other family members, such as fathers, mothers, sisters, and in-laws during decision-making. Most of the respondents (five mothers, four husbands, and four mothers-in-law (13 out of 19)) reported that the other family members shared their opinions and support regarding the physicians’ advice and sometimes provided monetary support. In one case, neighbors were involved and served the same purpose as family members. Those family members’ and neighbors’ involvement in the decision-making process varied, based on their availability and accessibility, and the urgency of the C-section decisions. Sometimes the respondents involved other physicians for a second opinion regarding the necessity of the C-section.

A 40-year-old mother described her family members’ support:

*I consulted with everyone, with my husband, parents, and sisters. They said, as you have to remove the tumor [in the uterus], you can save money by doing both in one operation, then do the Caesar … Suppose, if there was no tumor [in the uterus], then everyone would have advised for normal delivery, would have told to do the delivery at home. Because of that tumor, everyone around me, all of my relatives, told me to do the Caesar.*

Almost all the respondents reported that mothers had little to no say in the C-section decision-making process, and physicians primarily made the decisions with input from husbands and other relatives.

### 3.3. The Extent of Information Received Regarding C-Sections

#### 3.3.1. During ANC

All the respondents said that all the study mothers attended at least one ANC visit during pregnancy. During those ANC visits, pregnant women received information about their physical condition and nutritional requirements.

One 40-year-old mother-in-law said:

*I took my daughter-in-law many times, did the ultra (ultrasonography) two times, and had the checkup every month, did everything that needed to be done … Every time we went for a checkup, they examined whether the baby was well, whether the mother was in good condition.*

Eight out of 19 respondents (One husband, three mothers-in-law, and four mothers) were informed about delivering the baby through C-sections during the ANC visits. Half of those who received this information perceived history of previous C-sections as the absolute indication for subsequent C-sections. They did not mention any counseling about the choice of mode of childbirth.

The 40-year-old mother-in-law, whose second grandchild was delivered by C-section, reported that:

*The doctor said that the elder child was delivered by Caesar, so the younger one will also be delivered by Caesar … The doctors told that, as the previous childbirth was in Caesar, this time might need the same, so be prepared.*

Women were guided to prepare for elective C-sections. They did not mention receiving information regarding comparative risks and benefits of C-sections and NVDs, attending childbirth counseling sessions, or receive advice for the trial of labor. Only one respondent mentioned that she received information about C-section complications. The doctor explained to her the experiences she may have in the aftermath of a C-section, including difficulty in heavy work, and increased risk of a third C-section.

She remarked that:

*I went there (an NGO healthcare facility) with the doctor’s prescription, and they gave me the tablets of iron, calcium, vitamins, and gastric (anti-ulcer drugs), and I used to take them … They asked me, was your daughter born in normal delivery or Caesar? I replied, Caesar. Then they said, you will do the Caesar two weeks earlier … I knew that after doing Caesar, I would have a problem doing heavy works. It would strain a bit in place of Caesar cut, heavy work could not be done, and could not have more than three children.*

Moreover, some of the elective C-section mothers received assurance from the physicians that C-sections are safe procedures.

One 45-year-old mother-in-law told that:

*We didn’t ask the doctors about Caesar. On their own, they assured us by saying, why are you so afraid? There is nothing to fear.*

#### 3.3.2. During Emergency

Out of 19 respondents, 11 (four husbands, two mothers-in-law, three mothers, and two other family members) stated that mothers who had emergency C-sections were not informed during ANC visits about the possibility of a C-section in case of an obstetric emergency. According to them, providers never counseled them on the choice of mode of childbirth. One mother mentioned that she attended group sessions with other pregnant women in a healthcare facility of a non-governmental organization (NGO). During those sessions, she was encouraged to deliver in the facility. The respondent received information about the danger signs of pregnancy, but she did not receive counseling related to the choice of mode of childbirth in case of an obstetric emergency.

Among the 11 emergency C-section cases, three had a history of a trial of labor at home, and eight underwent trial in a health care facility. Respondents perceived prolonged labor or fetal distress as the main indication for C-sections. They said that the physicians only mentioned the necessity of the C-section but did not provide any detailed information regarding the indication. Most of the time, physicians convinced the respondents to deliver the baby via C-section by saying that there might be negative impacts on the baby. However, the respondents did not receive detailed information on these negative impacts. Decisions were made in haste and sometimes in the absence of husbands or in-laws. During the decision-making process, patients and their family members were not informed about the procedure or the risks. One husband was the primary decision-maker for a C-section of his second child. He expressed that not knowing about the complications of C-sections was the reason behind all their sufferings since his wife had undergone a C-section.

The 32-year-old husband remarked:

*My request to everyone is that, if possible, then do not go for Caesar. What I understand is that general people should not go for Caesar. The reason behind that is, we cannot keep someone to help with the household chores, we have to look after everything, including babies, and we have to maintain cooking too. But this is not possible for us because our economic condition is not good...In our case, we should not go for this.*

All the study mothers attended at least one ANC visit, but none reported receiving information regarding C-section indication, procedure, and comparative risks and benefits. Irrespective of the urgency of the C-section, the information asymmetry was prevalent.

### 3.4. Attitude towards the Informed Decision-Making Process

Upon asking respondents what they thought of receiving information related to C-sections during the antenatal period, such as indication, procedure, and risks/complications, only two husbands and three mothers responded. Almost all the respondents who shared their thoughts regarding shared decision-making shared a positive attitude towards the informed decision-making. Some respondents pointed out the importance of being informed about complications after C-sections and thought that the physicians should share that information when they advise women to go for C-sections.

One 20-year-old mother stated that:

*Of course, there is a need to know because no one knows how Caesar is done or the pain in administering the Caesar. Of course, we need to know … some have to do Caesar when they are in trouble.*

Four respondents (two husbands and two mothers) stated that knowing information about C-sections, including advantages and disadvantages, is critical before the procedure. It allows the mothers and their family members to prepare for the process, including financial resources, blood donor, and caregiver arrangements. The most commonly mentioned issues that respondents felt they needed to know before C-sections were the consequences of C-sections, advantages and disadvantages, and costs. Most respondents said that they did not need to know about the indication of C-section. They identified physicians and community health workers (CHWs) as a source of information.

A 27-year-old mother enthusiastically shared how CHWs could play a role in providing information regarding C-sections.

*There are BRAC center (an urban healthcare facility established by an NGO) and other centers. If we go there and want to know the information that we don’t know, they will surely inform us. That’s why we do not fear anything … For this reason, everything can be heard … they give us all the suggestions, so we don’t have to worry anymore.*

Some respondents (one husband, one mother, and three mothers-in-laws, 5 of 19 participants) did not feel the need to know more about C-sections during the antenatal period.

One 25-year-old mother mentioned that knowing everything about C-sections beforehand is not necessary. She said:

*I don’t think knowing about Caesar prior procedure is necessary … I don’t feel like knowing more. If Allah gives only one child to someone, then I would only pray that it lives well.*

The increased number of C-sections in the community made C-sections a normal occurrence for most respondents, and they perceived C-sections as risk-free procedures.

One 40-year-old husband said:

*We are seeing this for 10–15 years. Nowadays, no family goes for normal delivery; everyone chooses Caesar. Over time either the baby or the mother faces problems in normal delivery; got older seeing these. And so, we did not delay anticipating that the baby and the mother would be fine after Caesar.*

A 50-year-old mother-in-law’s comment echoed the previous statement of the husband and pointed out their feeling of safety with C-section delivery.

*Caesar has become very much normal these days, no more pain. Both the baby and pregnant women could live well.*

There were mixed opinions regarding the shared C-section decision-making. Some thought prior information about C-sections would help them better prepare to go through the process, while others thought the prior information about C-sections was unnecessary given the high prevalence of C-section in the study areas.

## 4. Discussion

Our exploratory study in a low-income urban community with low levels of education suggested that the decision-making process around C-section delivery was not shared decision-making in most cases, for both elective and emergency C-sections. The process for both emergency and elective C-section cases was similar, except for the time the decision was made. For elective C-sections, the decision was made at an earlier stage of the pregnancy compared to decisions made during obstetric emergencies in cases of emergency C-sections. In both cases, there was a lack of communication between the physicians and the patients and the family members of the patient. This study mirrored the findings of similar studies in Bangladesh that reported minimal communication between patients and physicians during C-section decision-making in healthcare facilities [37]. In 2012, a study reported that, in the Dhaka slums, for more than 50% of cases, the physician was the primary decision-maker for C-sections, and for 37% of cases, family members were also involved with C-section decision-making [38]. Patients usually rely on physicians’ knowledge, and they perceive the physicians to be in control of the final C-section decisions [37]. The flow of discussion in C-section decision-making revealed the lack of empowerment of the pregnant women. Limited involvement of pregnant women can be explained by other studies done in rural Bangladesh which reported that women are less empowered to decide their health care [39]. Only one in every eight women has been found to express having control over her health care decision-making [39]. Factors that can influence women’s empowerment include age, education, age of marriage, profession, and husband’s education [39].

Furthermore, some mothers and their family members did not think receiving detailed C-section information during the pregnancy period was necessary. This lack of demand for shared decision-making might be due to a lack of knowledge about shared decision-making and its importance in quality care. Interventions focused on improving knowledge about shared decision-making, its importance, and the rights of the patient and family members during decision-making may be an effective intervention strategy to enhance the demand for shared decision-making in the community.

In our study findings, information asymmetry related to C-sections was prevalent, similar to other LMICs [40]. One reason for this information asymmetry could be the format of ANC guidelines for Bangladesh, which lack counseling on the choice of mode of childbirth during ANC visits [41]. Researchers in high-income countries have leveraged this interaction between providers and pregnant women during ANC visits to test interventions to improve the shared decision-making related to C-sections and they found shared decision-making interventions significantly reduced decisional conflict during choosing the mode of childbirth and increased number of vaginal birth among the women with previous C-sections [42,43]. In Bangladesh, ANC visit rates are on the rise. In 2017, 82% of pregnant women received at least one, and 47% at least four ANC visits [44]. Thus, this ANC visit platform could be an opportunity for implementing shared decision-making for C-sections in Bangladesh.

In Bangladesh, a pluralistic healthcare system provides perinatal services at both community and facility-level delivered by the government, nongovernmental organizations, and private organizations [45]. ANC services are available both at the community and facility levels, however provider type, service quality, and cost may vary across facilities [45,46]. Emergency obstetric care services are usually available in sub-district, district, and specialized hospitals [45,47]. For 10,000 patients in Bangladesh there are only 5.6 doctors [48], and the number of nurses, midwives, and medical technologists is 40% and 24% of the number of doctors [45]. Insufficient human resources and a higher patient load are some of the main factors in Bangladesh affecting the healthcare service quality [37]. Some of the health system challenges that can affect the implementation of shared decision-making for C-sections during obstetric emergencies are inadequate staff and physicians for managing the patient load, lack of space in healthcare centers for providing care while maintaining patients’ privacy, and lack of evidence-based care in emergencies [37]. Another challenge could be the perception of C-sections by society as a safe procedure. Lack of demand by the pregnant women and family members for detailed C-section information during antenatal period can act as a barrier in improving the shared decision-making process in C-sections. Increasing this demand in the community could be challenging in LMICs, and a solution to overcome the challenge could be the availability of CHWs from different NGOs who can reach the population at community level to increase the awareness regarding the importance of shared decision-making during healthcare services, including C-sections. In Bangladesh, more than 4000 NGOs are working for marginalized populations [49]. Some of those NGOs provide maternal and child health care, along with other services [49]. In addition to the NGO services, over the last 10 years, the government of Bangladesh has taken the initiative to increase the number of midwives to improve the perinatal care services in Bangladesh [50,51]. An evaluation of a midwife-delivered community-based maternity care program in Bangladesh revealed lower maternal mortality in the areas where the midwives were available [52]. These existing resources of CHWs and midwives in addition to doctors, nurses, and other healthcare providers can be used to fill the gap of implementing shared decision-making for C-sections by reducing the information asymmetry in childbirth-related healthcare provision. To achieve this goal, a feasibility study using existing healthcare resources to implement a shared decision-making process could help the future program implementation related to the C-section decision-making.

### Strengths and Limitations

This study has several strengths, including exploring different household level decision-makers’ perspectives in the C-section decision-making process. We also explored the information women and family members received during the antenatal period. Finally, the respondent’s attitude towards shared decision-making helped us understand the existing knowledge and demand of shared decision-making in the slum population. The main limitations of this study are that we only sampled participants from urban slum populations and we did not examine the provider’s perspective, institutional capacity, and scope of implementing a shared decision-making process in Bangladesh’s current healthcare settings.

## 5. Conclusions

Overall, in the slums of Dhaka, Bangladesh, the C-section decision-making process did not fulfill the shared decision-making criteria, and women were not involved in all decision-making steps. Further studies should explore providers’ perspectives and institutional capacity to implement shared decision-making for C-sections. In LMICs, developing and implementing an intervention package to combat information asymmetry should consider the ANC coverage, availability of CHW and midwives, creation of demand in the community for shared decision-making, as well as existing barriers in the health system and the community. An effective, context-specific shared decision-making intervention has the potential to reduce the rate of unnecessary C-sections and related complications, while empowering pregnant women and their families.

## Data Availability

All the relevant data used to analyze the reported findings are available from the corresponding author on reasonable request. The data are not publicly available because we did not ask participants to consent to raw data sharing outside of the research team. Public sharing of the data could compromise anonymity and research participant’s consent.
